# Deformed wing virus variant shift from 2010 to 2016 in managed and feral UK honey bee colonies

**DOI:** 10.1007/s00705-021-05162-3

**Published:** 2021-07-17

**Authors:** J. L. Kevill, K. C. Stainton, D. C. Schroeder, S. J. Martin

**Affiliations:** 1 Department of Veterinary Population Medicine, University of Minnesota, 1365 Gortner Ave., Saint Paul, MN 55108 USA; 2 Department of Natural Sciences, Bangor University, Bangor, LL57 2DG Wales UK; 3 School of Environment and Life sciences, University of Salford, Manchester, M5 4WX UK; 4 The Pirbright Institute, Ash Road, Pirbright, Woking, GU24 0NF UK; 5 Fera Science Ltd., NAFIC, Sand Hutton, York, YO41 1LZ UK; 6 Environmental biology (Virology), Biomedical Sciences, University of Reading, Reading, RG6 6AH UK

## Abstract

Deformed wing virus (DWV) has been linked to the global decline of honey bees. DWV exists as three master variants (DWV-A, DWV-B, and DWV-C), each with differing outcomes for the honey bee host. Research in the USA showed a shift from DWV-A to DWV-B between 2010 to 2016 in honey bee colonies. Likewise, in the UK, a small study in 2007 found only DWV-A, whereas in 2016, DWV-B was the most prevalent variant. This suggests a shift from DWV-A to DWV-B might have occurred in the UK between 2007 and 2016. To investigate this further, data from samples collected in 2009/10 (n = 46) were compared to existing data from 2016 (n = 42). These samples also allowed a comparison of DWV variants between *Varroa*-untreated (feral) and *Varroa*-treated (managed) colonies. The results revealed that, in the UK, DWV-A was far more prevalent in 2009/10 (87%) than in 2016 (43%). In contrast, DWV-B was less prevalent in 2009/10 (76%) than in 2016 (93%). Regardless if colonies had been treated for *Varroa* (managed) or not (feral), the same trend from DWV-A to DWV-B occurred. Overall, the results reveal a decrease in DWV-A and an increase in DWV-B in UK colonies.

**Supplementary Information:**

The online version contains supplementary material available at 10.1007/s00705-021-05162-3.

## Introduction

Globally, honey bee colonies have been declining, with many reasons given, including pests and pathogens [1–4], pesticides [5, 6], habitat loss [7, 8], and the synergy between these factors [9]. Pathogens are identified as a major driver behind recent colony declines, in particular the association between the *Varroa destructor* mite (*Varroa*) and deformed wing virus (DWV) [10, 11]. DWV is a small (30 nm), single-stranded, positive-sense RNA virus belonging to the family *Iflaviridae* within the order *Picornavirales*. DWV diversity is reduced in the initial presence of *Varroa*, and one dominant master variant is selected [10, 12]. Prior to the introduction of *Varroa* to colonies of *A. mellifera*, DWV is typically absent or at very low prevalence and load, but with high viral diversity [10]. High DWV loads have been detected in over 55% of all colonies or apiaries surveyed across 32 countries [13]. Symptomatic DWV manifests with dead pupae and deformed wings, discolouration, and bloated abdomens in adult bees. Asymptomatic infected bees may have a reduced lifespan [14, 15], impaired learning and memory [16], precocious behaviour [17], and perturbed immunity [12]. At the colony level, large numbers of mites and DWV lead to colony collapse [15].

The DWV complex is a quasispecies and as such is comprised of a diverse population of variants. Three closely related master variants have been identified so far: DWV-A [18], DWV-B (Varroa destructor virus 1) [19], and DWV-C [20]. DWV-A has been linked to colony losses [10, 21], whilst there is contention in the literature about the effects of DWV-B on colony health. In 2015, long-lived healthy *Varroa*-resistant colonies were found only to be infected with DWV-B [22]. This suggested that DWV-B may offer protection to colonies, via a phenomenon known as superinfection exclusion (SIE) [22], where DWV-B outcompetes the virulent DWV-A [10, 23, 24]. Furthermore, DWV-B is present in healthy colonies [23] and in those in which overwintering colony losses are low [21, 24]. In laboratory experiments, DWV-naïve honey bee pupae injected with DWV-A were less likely to survive when compared to larvae injected with DWV-B [25]. However, DWV-B has been shown to be lethal when experimentally injected into adult bees [26], and has also been attributed to the loss of workers during the overwintering period [27]. Notably, the colonies in this study [27] nonetheless survived the overwintering period and were still viable the following spring. It was also observed in cage experiments that DWV-B was equal to DWV-A at causing pupal death and wing deformities [28]. DWV-C is rare in both the UK and USA [21], whilst it has been detected in stingless bee colonies in Brazil [29] and was found to be the dominant variant in small hive beetle (*Aethina tumida*) samples from Hawaii [30], suggesting that DWV variants may have favored hosts.

The differing outcomes of colony health due to the prevalence and abundance (viral load) of different DWV master variants leads to a requirement for longitudinal studies that provide continual monitoring of DWV variants and honey bee pathogens, especially before and after the overwintering period. These types of studies will help to identify trends in colony loss and survival. To date, comparisons of master DWV variant and year have been made in the USA, with an increase in DWV-B prevalence from 2010 to 2016 [31]. However, DWV-A remained the dominant USA variant and was linked to colony loss [21]. Analysis of English honey bee samples collected in Devon in 2007 revealed that colonies were mostly dominated by DWV-A, and DWV-B was absent [32]. A longitudinal study of a subpopulation of these Devon bees revealed that an increase in viral load of DWV-A and C was correlated with overwinter colony losses [24, 32]. A country-wide survey of the UK in 2016 found no difference in DWV load between spring and autumn [21], and also that DWV-B was by far the most dominant and prevalent DWV master variant [21]. This suggests that a similar change may be occurring in the USA, as it has over the last ten years in the UK. Furthermore, in 2009, a total of 389 honey bee and 95 mite samples from 32 geographic regions across four continents were analyzed [11]. Of these, 246 (63%) tested positive for DWV, 205 of which (83%) were DWV-A and 41 (17%) were DWV-B. Interestingly, in 2009, all DWV-B samples were from Europe (France, n = 25; Germany, n = 13; Romania, n = 2; UK, n = 1) and were the same recombinant, composed of DWV-B *Lp* and *Vp3* genes and DWV-A helicase and *RdRP* genes, and similar recombinants were also reported in the UK [33]. In France, a DWV-A/DWV-B recombinant with three breakpoints in the 5’ UTR, *Vp3* and helicase gene was found [34], when previous studies had only detected variants with one or two breakpoints. A recent study of *Apis mellifera* and *A. cerana* in Asia also identified DWV-A and DWV-B recombinants in the 5’ UTR, *Lp, Vp1, Vp2*, alongside recombinants of DWV-A and an unknown variant in the *Vpg* and *RdRp* genes [35] (Supplementary Fig. S1B).

To investigate the possibility that a variant shift from DWV-A to DWV-B has occurred in the UK, we screened both managed (*Varroa*-treated) and feral (*Varroa*-untreated) honey bee colonies from 2009/10 and compared them to colonies analyzed in 2016 [24].

## Materials and methods

### Sample collection

**Fig. 1:**
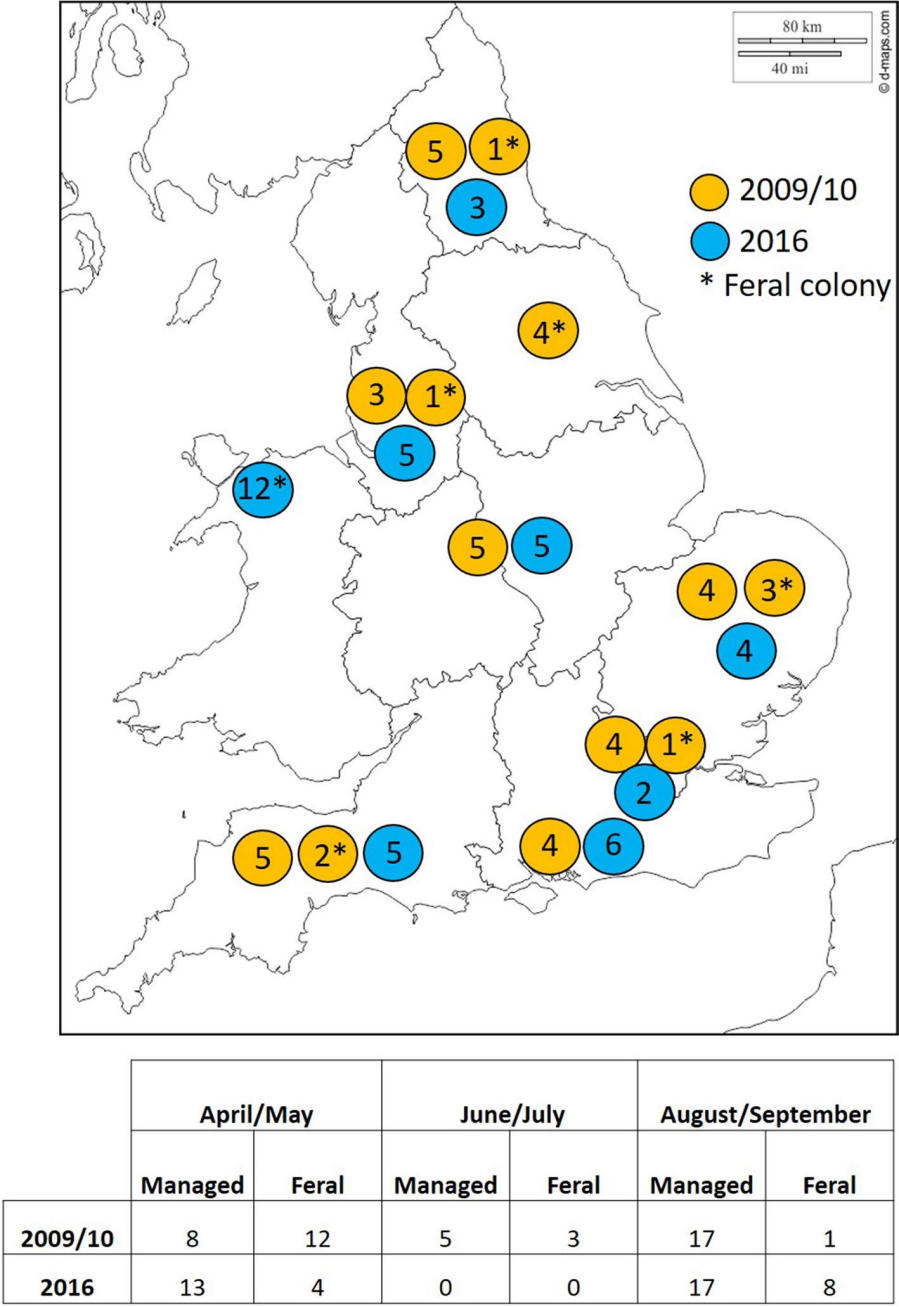
Regional map showing sampling locations. The map shows the region and number of colonies sampled by year. The 2009/10 colonies are yellow, the 2016 colonies are blue, and feral colonies are marked with *. Four feral samples are not listed on the map, as their location was unknown. The table at the bottom shows the number of samples taken for each group by year and month.

All colony samples contained approximately 30 adult bees, for both 2009/10 and 2016 samples. A total of 16 feral colonies from 2009/10 were kindly donated to this study by Catherine Thompson [36]. The 30 managed, *Varroa*-treated 2009/10 colonies were provided by the National Bee Unit (NBU). The 2016 samples were collected in England and Wales by beekeepers as part of a larger study conducted in 2016 [21]. The distribution of samples and month collected for 2009/10 and 2016 are shown in Figure 1. All feral colonies were from wild, free-living bees that had a history of >1 year at the nest site, as reported by Thompson et al. [36] and the Welsh beekeepers that took part in the 2016 study [21].

## Sample processing

A pool of 30 honey bees from each colony was homogenized using the mesh bag method [37] for samples collected in 2009/10, whilst a pool of 30 honey bees from 2016 was exposed to liquid nitrogen and homogenized using a mortar and pestle. RNA was extracted from a ~30-mg subsample from previously homogenized honeybees using an RNeasy Mini Kit (QIAGEN), following the manufacturer’s instructions. RNA was quantified using a NanoDrop spectrophotometer and were diluted to 50 ng/µl. We used the ABC assay [24], which reports on the conserved *RdRp* gene and represents the 3’ region of the DWV master genomes only. This assay cannot report on the prevalence of any DWV recombinant but rather provides an overview of whether there is a shift from DWV-A and its associated recombinants, and DWV-B and its associated recombinants, using a conserved region of the viral genome. Real-time PCR was performed using a Sensifast SYBR No-Rox One Step Kit (Bioline) with primers for DWV-A, DWV-B, DWV-C as described previously [24]. The RT-qPCR program is as follows: 45 °C for 10 min and 95 °C for 10 min, followed by 35 cycles of 95 °C for 15 s, 58.5 °C (DWV-A and DWV-B) or 61.5 °C (DWV-C) for 15 s, and 72 °C for 15 s. Each plate included an RNA standard curve and a no-template control. Actin controls were used to confirm the viability of the RNA from the NBU due to long-term storage of the samples. Melt curve analysis confirmed the absence of DWV in the no-template control and that one PCR product was amplified.

In addition to the ABC assay [24], a recombinant PCR assay [38] was performed on a subset of samples collected in 2016. Four combinations of DWV strain-specific primers (Supplementary Table S1) were used to amplify fragments >5 kb of DWV master variants and recombinants. Briefly, total RNA was used as a template to synthesise cDNA using an Invitrogen SuperScript IV First-Strand Synthesis System (Thermo Fisher Scientific) as per the manufacturer’s instructions. PCR reactions were prepared using a Q5 High-Fidelity DNA Polymerase Kit and 10 mM dNTPs obtained from New England Biolabs. Each reaction contained 5 µl of 5x Q5 buffer, 0.5 µl of 10 mM dNTPs, 0.25 µl of Q5 high-fidelity DNA polymerase, 1.25 µl of forward DWV-variant-specific capsid primer, 1.25 µl of reverse DWV-specific RdRp primer, 14.75 µl of nuclease-free water, and 2 µl of cDNA template. The thermocycler was set to initial activation at 98°C for 30 s, followed by 35 cycles of denaturation at 98°C for 10 s, annealing at 54°C for 20 s, and extension at 72°C for 3 min, followed by a final extension at 72°C for 2 min. Results were then visualised via gel electrophoresis using a 1.4% agarose gel containing gel red and a 1-kb hyper ladder.

## Analysis of the results

The viral load is expressed as genome equivalents per honey bee, using the following equations [24]:

1. Copy number RNA = (concentration RNA (ng/µL) × 6.022 × 10^23^)/(fragment length base pairs × 10^9^ × 325)
2. Genome equivalents = (average copy number generated by RT-qPCR) × (RNA dilution factor) × (elution volume of RNA) × (proportion of bee material*)

*Refers to the proportion of honey bee homogenate that was used to extract RNA (e.g., 30 mg homogenate / weight (mg) of a whole bee = the proportion of bee material).

The viral load of DWV variants was converted to a log_10_ value, and the total absence/presence and percentage of the DWV variant in each sample were determined. Any sample below 10^3^ genome equivalents is too low to be reliably quantified, and samples with no amplification or melt curve were considered negative. Statistical analysis was conducted using non-parametric tests, as a Shapiro-Wilk test revealed that the viral load data were non-normally distributed. A Kruskal–Wallis test was used to compare the viral loads of each DWV variant. Comparisons of the 2009/10 and 2016 data sets were made for all the colonies and also between the feral (*Varroa*-untreated) and managed (*Varroa-*treated) colonies. Only DWV-A and DWV-B data were used for analysis, as DWV-C was rare. Post-hoc analysis was conducted using Dunn’s test of multiple pairwise comparisons [39]. The significance threshold was set at *p* < 0.05, and the significance levels were adjusted when multiple comparisons were made using Bonferroni correction (significance threshold/number of comparisons). A proportion Z test was used to calculate differences between percentage data.

## Results

### DWV prevalence and viral load for all colonies sampled in 2009/10 and 2016

**Fig. 2:**
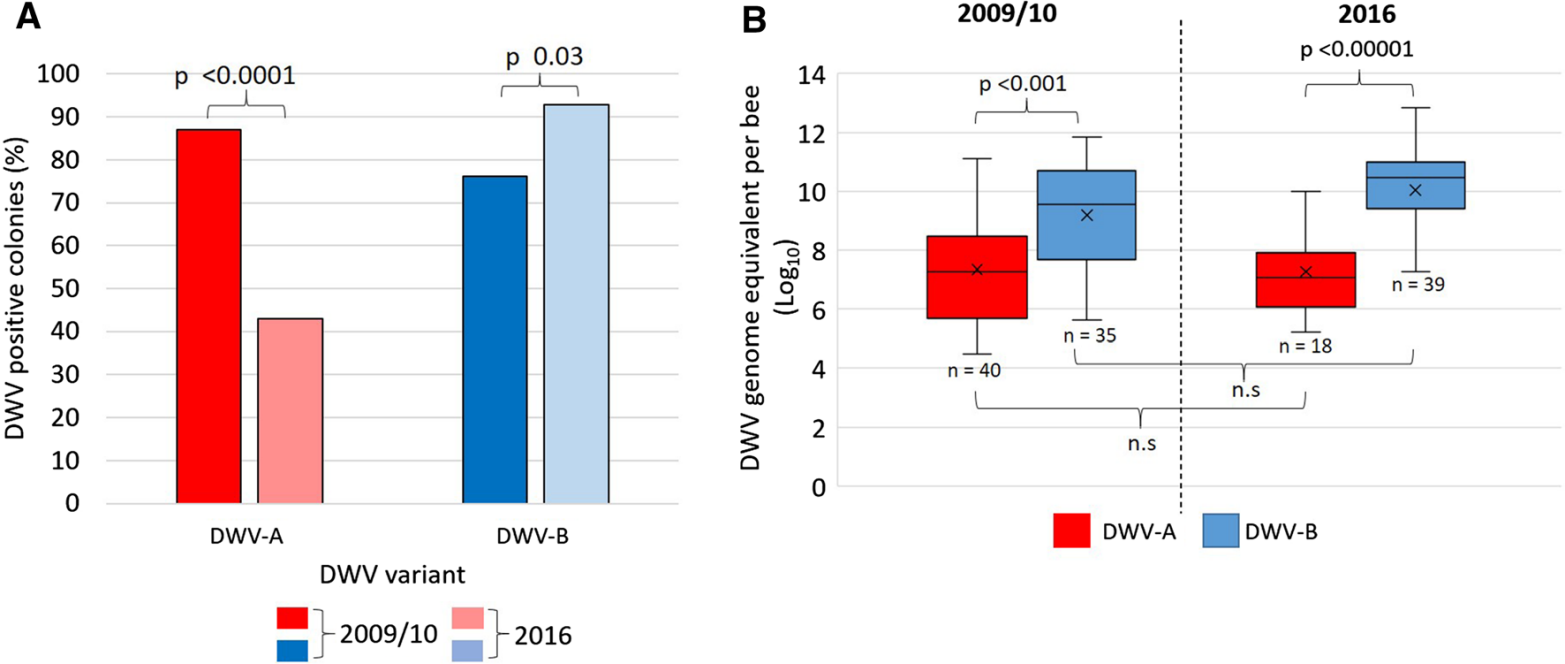
DWV prevalence and viral load in 2009/10 and 2016. (A) percentages of DWV-A- and DWV-B-positive colonies for the years 2009/10 and 2016. *p-*values were calculated using a proportional *Z* test. (B) Boxplots showing the DWV-A and B viral load of positive colonies for sample years 2009/10 and 2016. *p*-values were calculated using the Kruskal Wallis test and Dunn’s post-hoc test.

A significant reduction in DWV-A-infected colonies occurred from 2009/10 (87%, n = 46) to 2016 (43%, n = 42; Z score, 4.3595, *p* < 0.0001; Fig. 2A), whereas the prevalence of DWV-B-infected colonies increased significantly from 76% to 93% between the years 2009/10 and 2016 (Z score, -2.1483, *p =* 0.03; Fig. 2A). During the study, there was no significant difference in DWV-A or DWV-B viral load from 2009/10 to 2016. Significant differences occurred when comparing DWV-A to DWV-B for each year, as DWV-B was always present at higher viral loads than DWV-A (Dunn’s test; 2009/10, *p* < 0.001; 2016, *p* < 0.00001; Fig. 2B). A small subset of samples from 2016 were analysed for DWV recombinants (Supplementary data). DWV recombinants did not dominate the 2016 dataset and were often detected in the presence of a master variant (Supplementary Fig. S1 [38]).

## Regional DWV variant composition of managed colonies screened in 2009/10 and 2016

**Fig. 3:**
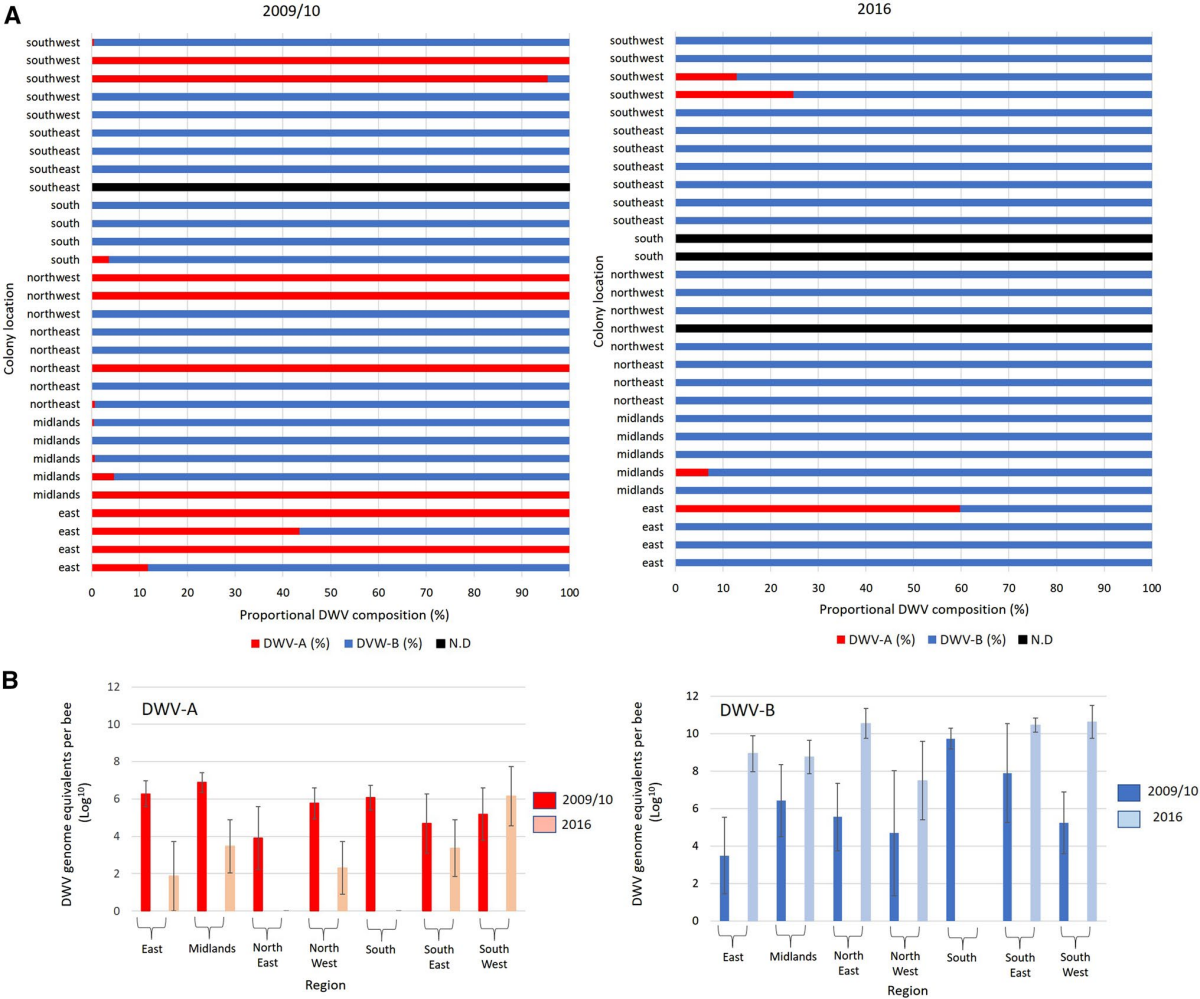
DWV composition in colonies. (A) location and proportional DWV composition (DWV-A, red; DWV-B, blue; not detected (N.D.), black) for each managed *(Varroa* treated) colony in 2009/10 and 2016. (B) The mean DWV-A and DWV-B viral load (log_10_), per region for all of the managed (*Varroa*-treated) colonies sampled in 2009/10 and 2016. Error bars show the standard error of the mean.

Individual analysis of each managed honey bee colony screened, per region, revealed that whilst DWV-B was the dominant variant for both years, DWV-A was far more prevalent in 2009/10 than in 2016 (Fig. 3A). A total of eight colonies were dominated by DWV-A in 2009/10, compared to only one colony in 2016 (Fig. 3A). The proportion of DWV-A in the 2016 colonies was less than 1% for six colonies and therefore cannot be visualised on the histogram (Fig. 3A), while the prevalence was 7% for one colony and >10% for three colonies (Fig. 3A). When comparing mean DWV loads in each region, DWV-A was >tenfold higher in samples collected in 2009/10 than in 2016 in all regions, whilst the DWV-B viral load increased >tenfold between 2009/10 and 2016 everywhere except in the South of England (Fig. 3B).

## DWV variant load and prevalence in feral and *Varroa*-treated (managed) colonies from 2009/10 to 2016

**Fig. 4:**
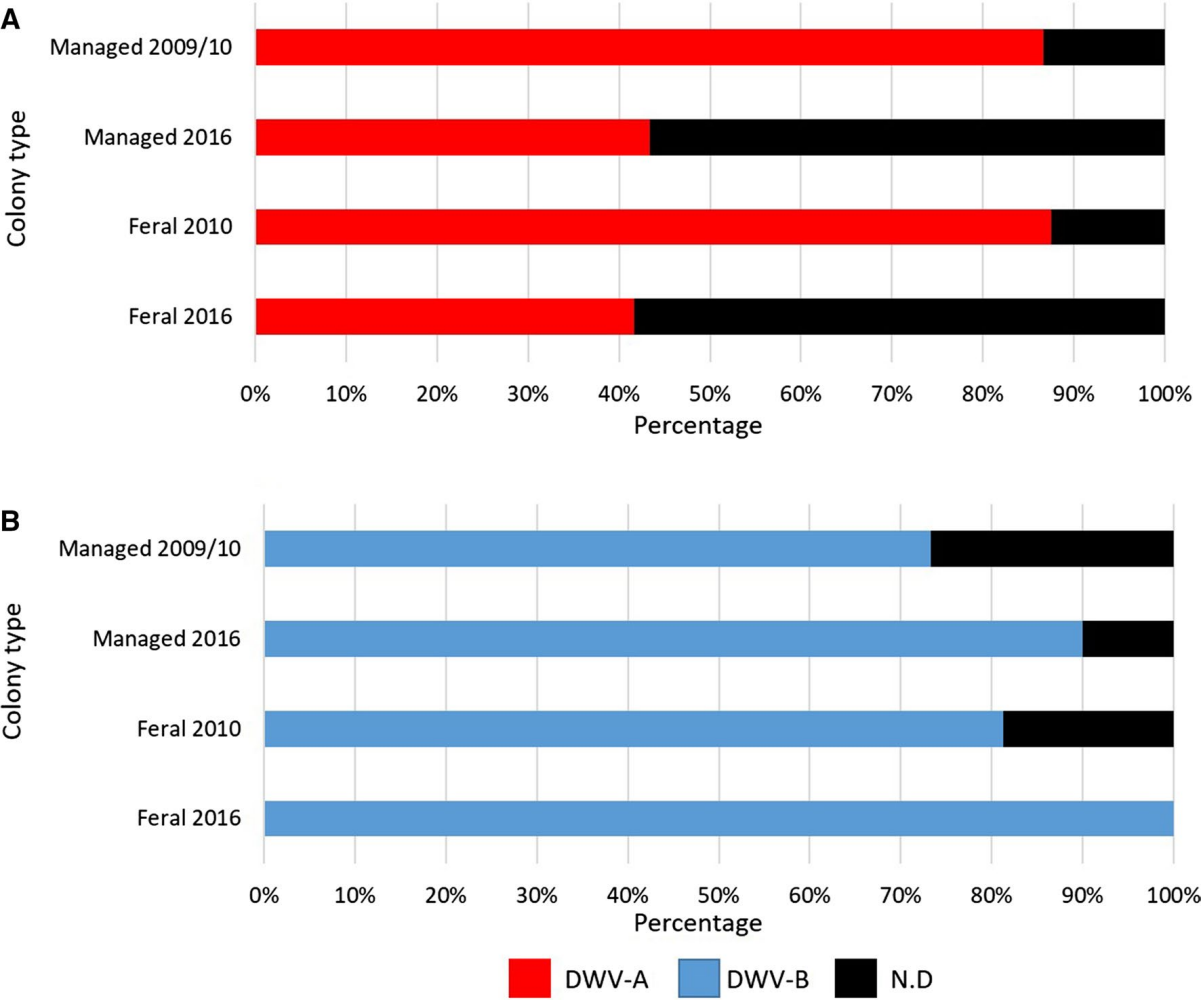
DWV variant abundance in managed and feral colonies. (A and B) Comparison of the prevalence of (A) DWV-A- and (B) DWV-B-positive feral and managed colonies sampled in 2009/2010 and 2016

**Fig. 5:**
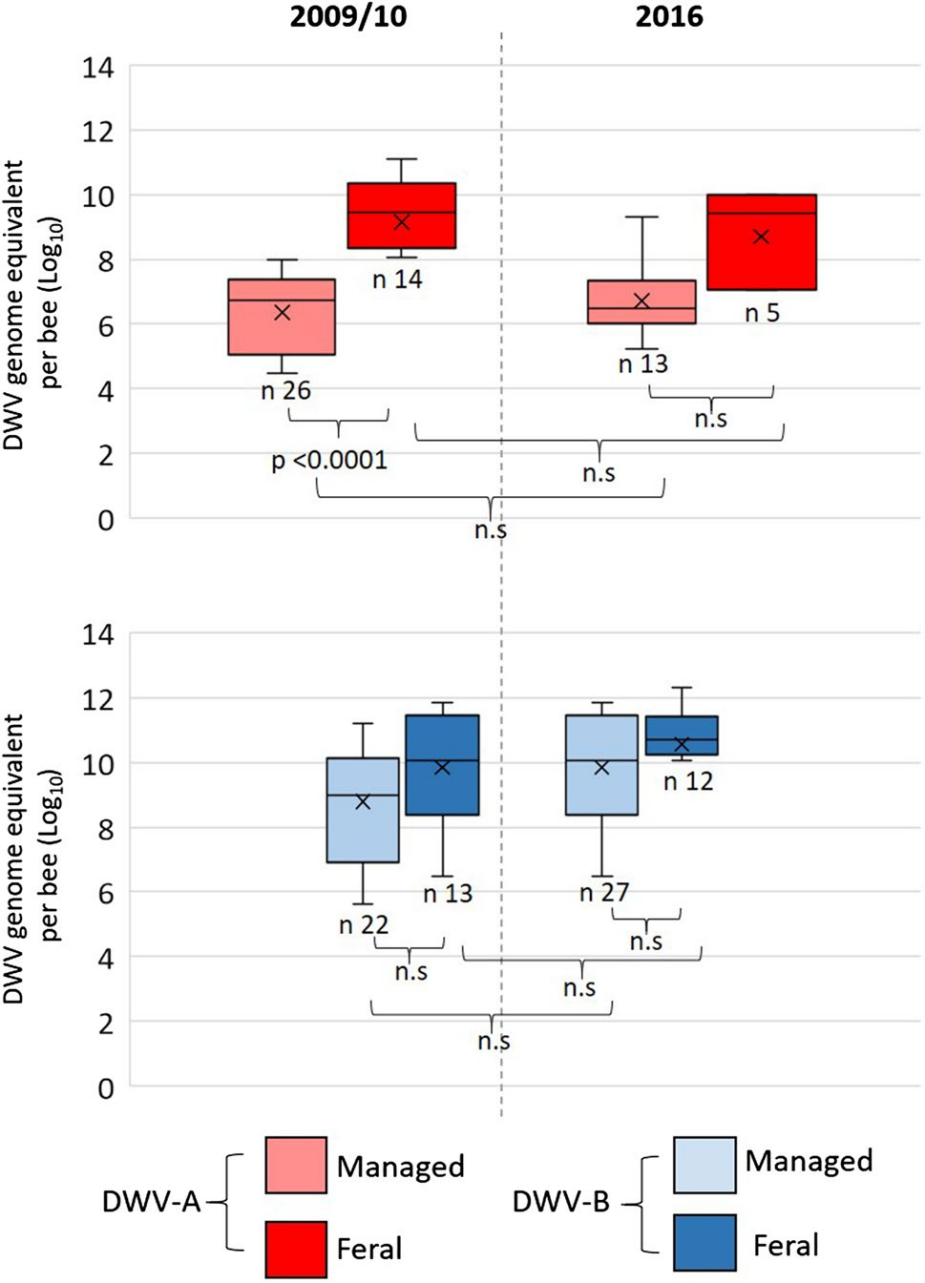
DWV load in feral versus treated colonies. A comparison of DWV-A and DWV-B viral loads (log_10_ DWV genome equivalents per bee) in feral and treated colonies from 2009/10 and 2016 is shown. Statistical comparisons were performed using the Kruskal-Wallis test and Dunn’s post-hoc test using Bonferroni correction. The samples sizes (n) are also given.

The same pattern of DWV-A prevalence was observed in both *Varroa*-treated (managed) and feral colonies, as DWV-A prevalence decreased from 2009/10 to 2016 (Fig. 4). Interestingly, almost 87% and 88% of managed and feral colonies, respectively, were DWV-A positive in 2009/10, whilst this was reduced by half in 2016 (Fig. 4). The opposite trend was seen in both managed and feral colonies for DWV-B. In 2009/10, 73% and 81% of managed and feral colonies were DWV-B positive, respectively. This increased to 90% in managed colonies and 100% in feral colonies in 2016 (Fig. 4). The DWV viral load between years remained consistent regardless of management or year (Fig. 5). However, 2009/10 feral colonies had a significantly higher DWV-A load than the 2009/10 managed colonies (Dunn’s test; *p* < 0.0001; managed, 1 × 10^5^ – 1 × 10^8^; feral, 1 × 10^8^ – 1 ×10^11^; Fig. 5).

## Discussion

In this study, we detected a DWV master variant shift from DWV-A to DWV-B between 2009/10 and 2016, occurring over a similar period to that in the USA [31]. We found that, between 2009/10 and 2016, DWV-A prevalence decreased, whilst DWV-B prevalence increased. The switch from DWV-A to DWV-B may be explained by the ability of DWV-B to replicate to higher loads than DWV-A [25, 26, 28, 40], which accounts for why DWV-B was detected at higher loads than DWV-A for both sample years. When coinfection with both variants occurs, the DWV-A viral load is initially higher than that of DWV-B, but at 72 hours post-inoculation, DWV-B replicates to higher loads [25]. Superinfection exclusion (SIE) occurs when a pre-existing viral infection prevents a secondary infection with a closely related virus [41]. SIE has been demonstrated for DWV-B, but only at the colony level, as it is yet to be proven if DWV-B can exclude DWV-A from cells or at the individual bee level [25, 28]. The detection of DWV-A/DWV-B recombinants [34] provides evidence that DWV-B cannot exclude DWV-A from entering the cell. However, at the colony level, DWV-B reaches saturation in populations. This may be because it is less lethal to pupae than DWV-A [25] and replicates to a higher load. However, it should be noted that the presence of other viruses may have confounded this result, as black queen cell virus (BQCV) was detected in the DWV-A inoculum in the cited study [25]. In another study in which the inocula did not contain any viruses other than DWV-A or DWV-B, pupal mortality was equal regardless of DWV variant or DWV coinfection. However, those injected with DWV-B had a higher viral load as adults than those injected with DWV-A [28]. Rather than excluding multiple infections within the host, it seems to be more likely that DWV-B competes with and gradually replaces DWV-A at the colony level.

The ability of DWV-B to replicate to higher loads than DWV-A provides a possible evolutionary advantage over DWV-A and may be an example of viral attenuation, since longer-term trends indicate *Varroa*-virus-associated overwintering losses may be declining [21, 42, 43]. Furthermore, evidence suggests that DWV-B is able to replicate in *Varroa* mites, while DWV-A cannot [44], and this may play a role in the increased prevalence of DWV-B in colonies. In the currently DWV-A-dominated USA [21, 31], the rate of decline is periodically higher than in other temperate regions [45]. Therefore, it is expected that, over time, DWV-A would lose dominance in honey bee populations, as evidenced here within. It is expected that colonies in the USA [21, 31] will eventually also switch to DWV-B dominance. Since DWV-A was not completely eradicated from the English honey bee colonies sampled, a re-emergence of DWV-A may occur, highlighting the need for long-term landscape level monitoring over time.

Mutation rates in RNA viruses are high, and a few single amino acid changes can alter the pathogenicity of a virus [46]. Nucleotide differences in the internal ribosome entry site (IRES) structures of DWV-A and DWV-B may provide DWV-B an evolutionary advantage over DWV-A [25]. The IRES secondary structure is an important feature for many RNA viruses, as IRES elements are used to initiate translation and the replication processes [47, 48]. The IRES elements of DWV-A and DWV-B are located at the 5’ end of the genome, and the secondary structures are predicted to be the same for both variants [49]. DWV-A and DWV-B have 84% nucleotide and 95% amino acid sequence identity [19] across the whole genome; however, comparison of the first 818 nucleotides shows a 65% sequence identity between DWV-A and DWV-B in the region where the IRES is located [49]. Mutations in the IRES have been shown to alter the virulence and replication efficiency of RNA viruses [50–52]. It is possible that evolution has favourably altered the success of DWV-B, allowing for this genotype to persist and outcompete or suppress other DWV variants in populations of honey bees. We propose that recombination is an important mechanism for viral variant switching, and there is some evidence in the literature to support this idea [11], since the earliest recorded recombinants were those between the structural genes of DWV-B and the non-structural genes of DWV-A. This study reports on a small region (*RdRp*) of the DWV genome and therefore does not include the detection of A/B recombinants; however, a subset of samples from 2016 for which recombination data exist has been provided in the supplementary data (Supplementary Table S2 and Supplementary Fig. S1).

In order for a *Varroa* mite to complete its life cycle, it must enter an occupied brood cell, where it will reproduce and feed on the honey bee haemolymph [53] and fat body tissue [54]. If *Varroa* vectors a pathogen that is deadly to the host, the mite and its progeny die too. By comparing feral *Varroa*-untreated and managed *Varroa*-treated colonies, we were able to investigate whether the mite influenced which DWV variants were detected. Although we cannot confirm the complete absence of *Varroa* in the treated managed colonies, feral colonies are expected to have higher *Varroa* levels. It was speculated that a virulence trade-off must occur between *Varroa*, DWV, and the host for DWV populations to be maintained in the environment; however, vector-borne virulence trade-offs are hard to prove [55]. We found a similar level of prevalence of DWV-A and DWV-B in feral and managed colonies in both sample years, although feral colonies had a higher prevalence of DWV-B. No significant differences were recorded when comparing the DWV-B load in feral and managed colonies for both years. The DWV-A level was significantly higher in the 2009/10 feral colonies than in the 2009/10 managed colonies. It was found that management did not influence which DWV variant was present; the only difference observed was an increase in the proportion of DWV-B positives between feral and managed colonies. In addition, previous work conducted on these samples showed that the season (spring/late autumn) had no bearing upon DWV load or DWV prevalence [21], although increasing *Varroa* numbers have been shown to increase the DWV load. It has been demonstrated recently that the DWV-A load increases along with *Varroa* population growth, whilst *Varroa* numbers do not influence DWV-B load [56]. Therefore, it is entirely possible that the bees themselves are maintaining DWV-B infections within the colony. More colony-level studies are required to understand virulence trade-offs and the factors that influence them, as this type of study is lacking in the available scientific literature.

So far, DWV-B dominance in honey bee colonies may be beneficial, as DWV-B-infected colonies appear to be healthier than those infected with DWV-A [23], indicating that the disease induced by DWV-B may be lessening in severity naturally. The exact mechanism underlying the advantageous nature of DWV-B over DWV-A has yet to be identified; however, we demonstrate that DWV-A prevalence was dramatically reduced from 2009/10 to 2016, which coincided with an increase in DWV-B. This may be explained by genetic variations between the DWV master variants that favor host cell invasion and replication, as well as a potential tradeoff in DWV virulence initiated by the *Varroa* vectoring cycle.

## Supplementary Information

Below is the link to the electronic supplementary material.

Supplementary file1 (DOCX 192 KB)

Supplementary file2 (JPG 212 KB)
