# Supplementary material for: Deformed wing virus variant shift from 2010 to 2016 in managed and feral UK honey bee colonies
Source: Arch Virol. 2021 Jul 17;166(10):2693–702. doi: 10.1007/s00705-021-05162-3 (PMC8421296; doi:10.1007/s00705-021-05162-3)
Supplement: Supplementary file 1 — Supplementary file1 (DOCX 192 KB) [file 705_2021_5162_MOESM1_ESM.docx]

**Supplementary data**

A subset of UK samples collected in 2016 were screen via PCR to identify major DWV variants and recombinants; full details are published in [1]. Four combinations of DWV strain specific primers (Supplementary table S1) were used to amplify fragments >5 kb of DWV master variants and recombinants. Briefly, total RNA was synthesised into cDNA using an Invitrogen SuperScript IV first strand synthesis system (ThermoFisher Scientific), as per the manufacturer’s instructions. PCR reactions were prepared using a Q5 high-fidelity DNA polymerase kit and 10mM dNTPs obtained from New England Biolabs. Each reaction contained, 5 µl 5 x Q5 buffer, 0.5 µl 10mM dNTPs, 0.25 µl Q5 high fidelity DNA polymerase, 1.25 µl forward DWV variant specific capsid primer, 1.25 µl reverse DWV specific RdRp primer, 14.75 µl nuclease free H2O, and 2 µl cDNA template. The thermocycler was set to: initial activation 98^o^C for 30 secs, followed by 35 cycles of denature at 98^o^C for 10 secs, annealing at 54^o^C for 20 secs, extension at 72^o^C for 3 mins, followed by a final extension at 72^o^C for 2 mins. Results were then visualised via gel electrophoresis using a 1.4% agarose gel containing gel red against a 1kb hyper ladder.

Supplementary Table 1. Primers used to amplify >5Kb DWV fragments of master variants A and B, and recombinants thereof.

| Primer | Name | Sequence (5′–3′) |
| --- | --- | --- |
| Capsid A forward | A_Capsid_F2_AH | TTTGTGGAGCAAAGAATTGA |
| RdRp A reverse | A_RdRp_R_JK | CTCATTAACTGTGTCGTTGAT |
| Capsid B forward | B_Capsid_F2_AH | CAAGACAACTTGCTCAGCAT |
| RdRp B reverse | B_RdRp_R_JK | CTCATTAACTGAGTTGTTGTC |

Supplementary Table 2. Shows the DWV variant detected per colony, confirmed via gel electrophoresis. A heat map is provided to show intensity of band generated, orange boxes with red ++ indicate a bright band, whilst blue boxes with + indicate a band of lesser intensity. The – indicates a negative, and M refers to samples that have no data due to lack of RNA.

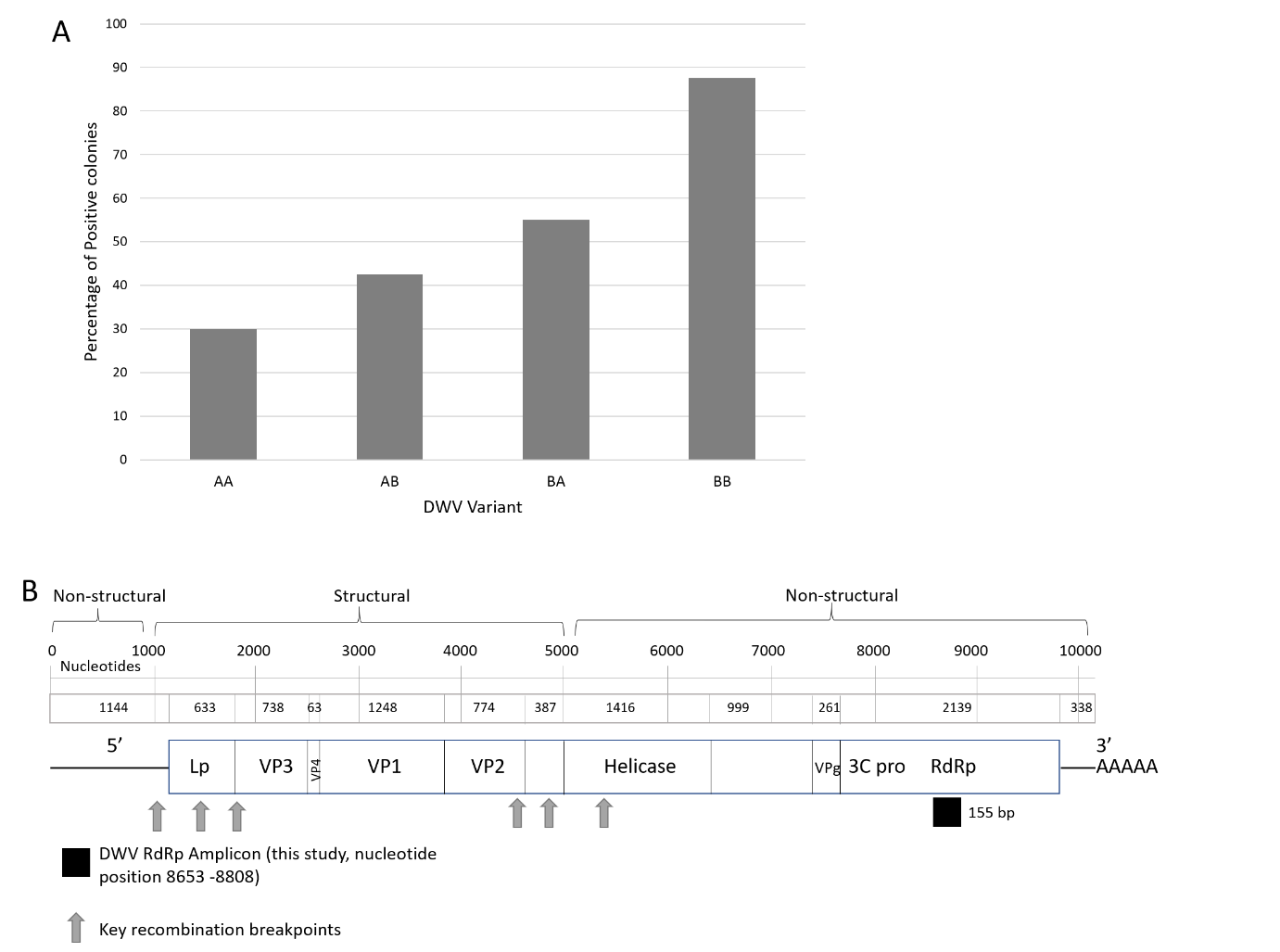


Supplementary Figure 1. A) percentage of 2016 colonies (n = 48) that had full length DWV-A, DWV-B and recombinants of DWV-A and DWV-B. B) shows the DWV genome [2], nucleotide scale and number of nucleotides encoding for each protein. The black box highlights the 155bp region amplified using the ABC assay [3], and arrows show key recombinant break positions within the DWV genome [4,5,6].

**References**

1. Kevill, J. (2019). *The role of resistance to Varroa destructor and Deformed wing virus in the European honey bee (Apis mellifera)* (Doctoral dissertation, University of Salford).
2. de Miranda JR, Genersch E (2010) Deformed wing virus. J Invertebr Pathol 103:S48-S61. doi:10.1016/j.jip.2009.06.012
3. Kevill JL, Highfield A, Mordecai GJ, Martin SJ, Schroeder DC (2017) ABC Assay: Method Development and Application to Quantify the Role of Three DWV Master Variants in Overwinter Colony Losses of European Honey Bees. Viruses-Basel 9 (11). doi:ARTN 31410.3390/v9110314
4. Ryabov EV, Wood GR, Fannon JM, Moore JD, Bull JC, Chandler D, Mead A, Burroughs N, Evans DJ (2014) A Virulent Strain of Deformed Wing Virus (DWV) of Honeybees (*Apis mellifera*) Prevails after *Varroa destructor*-Mediated, or In Vitro, Transmission. Plos Pathog 10 (6). doi:ARTN e100423010.1371/journal.ppat.1004230
5. Dalmon A, Desbiez C, Coulon M, Thomasson M, Le Conte Y, Alaux C, Vallon J, Moury B (2017) Evidence for positive selection and recombination hotspots in Deformed wing virus (DWV). Sci Rep-Uk 7. doi:ARTN 4104510.1038/srep41045
6. Fei DL, Guo YX, Fan Q, Wang HQ, Wu JD, Li M, Ma MX (2019) Phylogenetic and recombination analyses of two deformed wing virus strains from different honeybee species in China. Peerj 7. doi:ARTN e721410.7717/peerj.7214
