# Supplementary figures and images for: Deformed wing virus variant shift from 2010 to 2016 in managed and feral UK honey bee colonies
Source: Arch Virol. 2021 Jul 17;166(10):2693–702. doi: 10.1007/s00705-021-05162-3 (PMC8421296; doi:10.1007/s00705-021-05162-3)

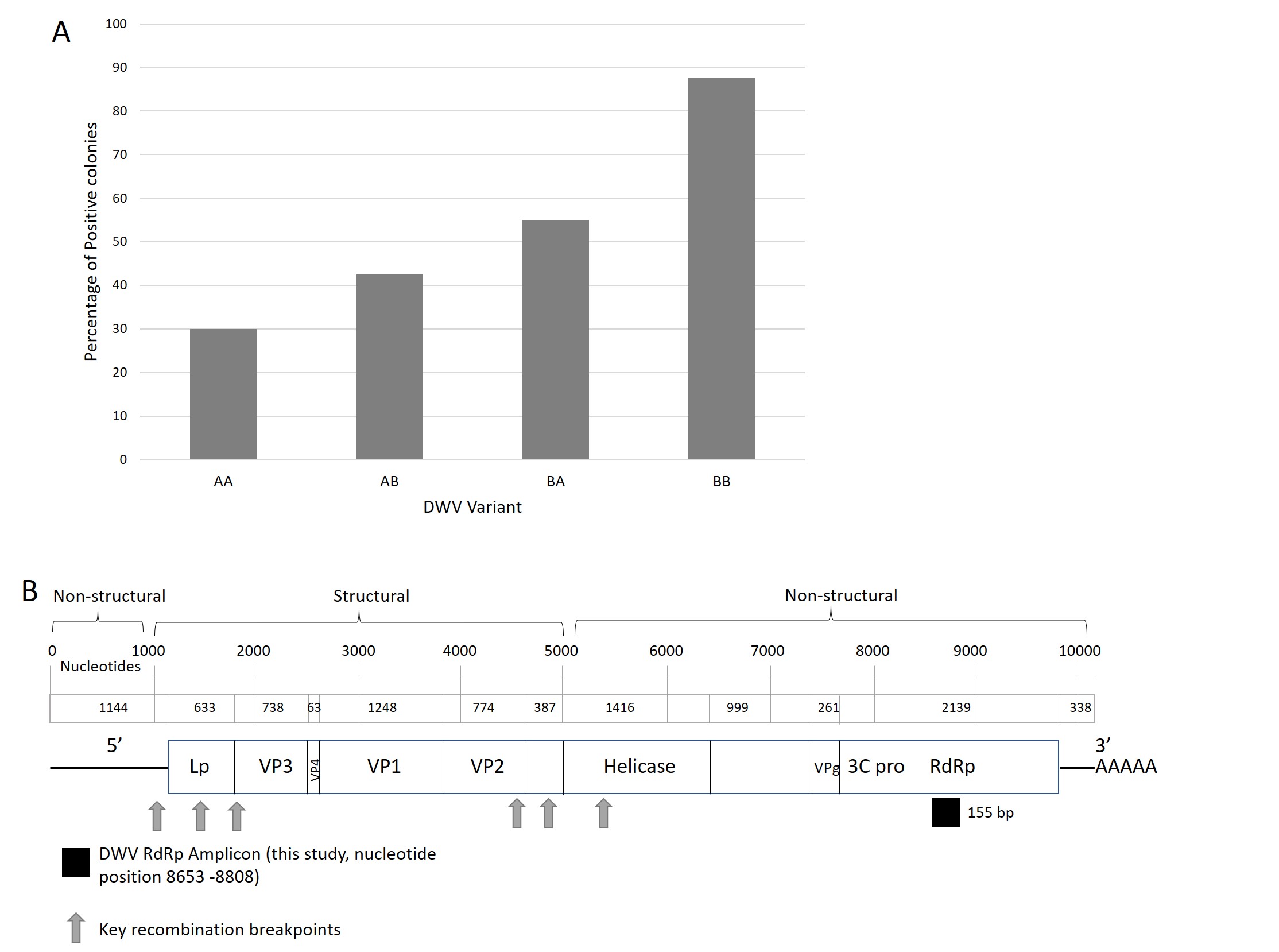

Supplement: Supplementary file 2 — Supplementary file2 (JPG 212 KB) [file 705_2021_5162_MOESM2_ESM.jpg]
